# Increased detection of Shiga toxin-producing *Escherichia coli* (STEC) O26: Environmental exposures and clinical outcomes, England, 2014–2023

**DOI:** 10.1017/S0950268825100654

**Published:** 2025-10-07

**Authors:** Lucy Findlater, Orlagh Quinn, Amy Douglas, Clare Sawyer, Victoria J. Hall, Claire Jenkins, Sooria Balasegaram

**Affiliations:** 1 UK Field Epidemiology Training Programme, UK Health Security Agency, London, UK; 2 Epidemic and Emerging Infections, UK Health Security Agency, London, UK; 3 National Institute of Health Research (NIHR), Health Protection Research Unit in Gastrointestinal Infections, University of Liverpool, Liverpool, UK

**Keywords:** Shiga-Toxigenic Escherichia coli, Epidemiology, Haemolytic uraemic syndrome, Farms, Nursery schools

## Abstract

In England, Shiga toxin-producing *Escherichia coli* (STEC) serogroup O26 has recently emerged as a public health concern, despite fewer than half of diagnostic laboratories in England having the capability to detect non-O157 STEC. STEC O26 cases frequently report exposure to farms or nurseries. We describe the epidemiology of STEC O26 and examine evidence for a relationship between O26 and exposure to these settings. We analysed national surveillance data describing laboratory-confirmed STEC cases and public health incidents over the past 10 years to explore the incidence, clinical outcomes, and association with farms and nurseries for STEC O26 cases compared to STEC O157 and other serogroups. Between 2014 and 2023, the proportion of STEC notifications which were STEC O26 increased from 2% (19/956) to 12% (234/1946). After adjusting for age, we found no difference in the likelihood of farm or nursery attendance between O26 and O157 cases but a significantly higher risk of HUS in O26 (adjusted risk ratio 3.13 (2.18–4.51)). We demonstrate that STEC O26 is associated with the same risk of farm or nursery attendance as other STEC serogroups but a higher risk of severe morbidity. Our findings reinforce the need for improved surveillance of non-O157 STEC.

## Introduction

Shiga toxin-producing *Escherichia coli* (STEC), also known as Vero cytotoxin-producing *E. coli* (VTEC), are zoonotic bacteria characterized by either or both of the *stx1* and *stx2* genes encoding the Shiga toxin [1]. Symptoms in humans range from gastroenteritis and bloody diarrhoea to severe complications such as haemolytic uraemic syndrome (HUS), a life-threatening condition associated with acute renal failure which occurs more commonly in children [2]. Worldwide, STEC infections are estimated to cause more than 1 million illnesses, 128 deaths, and nearly 13,000 disability-adjusted life years annually [3].

People can become infected with STEC by consuming contaminated food or water, through contact with animals and their environment including faeces, or through person-to-person transmission [4]. A wide range of farm and domestic animals act as reservoirs for STEC, including cattle, goats, and sheep [5, 6]. Outbreaks of STEC have been associated with exposure to farms, including petting farms where visitors may feed and handle animals [7–9]. Other high-risk settings for outbreaks of STEC are children’s nurseries, which are facilities commonly used in the UK for day care and education of children aged 0 to 5 years before they start school, where there may be preparation and provision of food and support with nappy (diaper) changing. Young children have a higher risk of STEC associated with their immature immune systems and underdeveloped personal hygiene practices, and there is opportunity for person–person transmission at nursery settings [10, 11].

Historically, the most commonly detected serogroup of STEC, which causes illness in England, was O157 [12]. However, non-O157 serogroups of STEC also cause severe disease and are increasingly recognized as a significant cause of infectious gastrointestinal (GI) illness internationally [13–15]. In England, culture methods at frontline diagnostic laboratories focus on the detection of STEC O157 [16]. Molecular methods, such as polymerase chain reaction (PCR) assays, can detect *stx* genes present in all STEC serogroups and are required to reliably detect non-O157 STEC in faecal specimens. Semi-selective culture media, such as sorbitol MacConkey agar and CHROMagar STEC™ facilitate the culture of STEC O157 and some (but not all) other serogroups from faecal specimens that test PCR positive for *stx* genes, but CHROMagar STEC™ is not yet widely used at frontline laboratories [17]. PCR testing was initially adopted by a small number of laboratories in England in 2013 and has become increasingly widespread, rising from 20% in 2018 to approximately 40% of diagnostic microbiology laboratories in 2023 using molecular diagnostic assays for GI pathogens [16, 18–22]. As not all laboratories use PCR for GI diagnostics, surveillance of non-O157 STEC in England is inconsistent. The epidemiology, health impact, and clinical and public health burden of non-O157 STEC remains unclear [18, 21, 23].

As PCR methods have improved detection of non-O157 STEC, STEC O26 has emerged as the most commonly identified serogroup after O157 in England, with incidence of O26 continuing to rise annually [16]. Outbreaks of STEC O26 associated with farm and nursery settings have been reported in recent years, in England and internationally, with previous surveillance data suggesting increasing numbers of farm and nursery associated STEC outbreaks in England [21, 24–26].

We analysed national surveillance data to describe cases and public health incidents (clusters, outbreaks, or public health investigations of at least one case) of STEC O26 over the past 10 years, in England. We explored the incidence, health impact, and evidence of any association with farms and nurseries for STEC O26 compared to STEC O157 and other serogroups.

## Materials and methods

### STEC surveillance

In England, all stool samples from hospitalized patients and community-acquired GI infections are tested for STEC O157 using cefixime-tellurite sorbitol MacConkey agar (CT-SMAC). Non-sorbitol fermenting colonies agglutinating with *E. coli* O157 antisera should be referred to the Gastrointestinal Bacterial Reference Unit (GBRU) at the UK Health Security Agency (UKHSA) for confirmation and typing [21]. At local laboratories which have also implemented commercial PCR testing targeting *stx*, when a faecal specimen tests positive for *stx*, a sample should be referred to GBRU for further testing, including a PCR test which specifically detects STEC O26 and culture [18]. Laboratories which do not have PCR diagnostics may send faecal specimens directly to GBRU for O26-PCR testing and further non-O157 testing. Referral of samples to GBRU is especially encouraged for children and those with bloody diarrhoea and HUS [27].

In England, STEC is a notifiable infectious disease under Schedule 2 of the Health Protection (Notification) Regulations (2010), and as such, medical practitioners and diagnostic laboratories are legally required to notify UKHSA when identified [28]. In England, following the reporting of a case, STEC operational guidance recommends that enhanced surveillance questionnaires (ESQs) are administered to cases, prioritized based on risk factors for severe infection, such as the presence of *stx2, eae*, or *aggR* genes, serogroup O157, as well as the age and clinical profile (bloody diarrhoea or HUS) of the case [23]. After the implementation of O26 PCR testing at GBRU, surveillance of STEC O26 was improved by the earlier detection of these cases and more timely completion of questionnaires [23, 27].

### Data collection

A dataset of all laboratory-confirmed STEC cases residing in England was obtained as a bespoke extract from the National Enhanced Surveillance System for STEC (NESSS) infection in England. NESSS was implemented in January 2009 and is maintained by the Gastrointestinal Infections, Food Safety, and One Health division at UKHSA. NESSS contains clinical, epidemiological, and microbiological data for all laboratory-confirmed STEC cases residing in England, from ESQs and from laboratory reports from diagnostic laboratories and GBRU [18]. All case records from NESSS with a sample date (or onset date, laboratory receipt date, or laboratory report date, if unavailable) from 1 January 2014 to 31 December 2023 inclusive were extracted.

Public health incidents were defined as clusters, outbreaks, or investigations of single settings linked to at least one case of STEC and reported to UKHSA. The investigation of STEC cases and reporting of regional public health incidents is prompted by local environmental health and UKHSA health protection teams. Regional STEC public health incidents handled by health protection teams are documented on the incident management system HPZone (national STEC incidents are not always recorded on this system) [29]. All STEC public health incidents on HPZone reported from 1 January 2014 to 31 December 2023 inclusive were extracted.

### Descriptive analysis

Data from 2014 to 2023 were analysed for trends in the number of STEC cases and in the incidence of STEC O26, STEC O157 and other serogroups. The other serogroups were grouped into one ‘other’ category due to the large diversity of non-O26, non-O157 STEC serogroups and the low numbers of individuals with each serogroup other than O157 and O26 [22]. Proportions of demographic factors, exposures, clinical symptoms, and microbiological characteristics amongst STEC cases were also calculated. Region was categorized using the nine geographic regions of England [30]. When examining nursery or farm-linked cases, exposure was defined as attendance at a farm or a nursery setting in the 7 days before onset of symptoms. Attendance at a farm included cases who had visited a farm or lived on a farm. Attendance at a nursery included nursery-age children and adults who worked at a nursery. Attendance at farms and nurseries was not mutually exclusive and some cases attended both settings; therefore, farm and nursery attendance were analysed separately.

Trends in the number of STEC public health incidents and the number which referred to STEC O26, STEC O157, both, or neither, were presented. Trends in public health incidents reporting a farm or nursery setting were also analysed. Public health incidents of STEC O26 and STEC O157 were defined as incidents in which reporting of ‘O26’, ‘O157’, or both could be identified within the descriptive notes. Manual review was conducted to remove public health incidents where ‘not O157’ or ‘not O26’ was written. Data were not available to identify the serogroups of individual cases within the incidents who may have had other or unknown serogroups. Farm or nursery setting was determined from manual checking of the incident descriptive notes.

### Regression analysis

Using data from 2019 to 2023, regression analysis was conducted to explore the association between STEC O26 and health outcomes and STEC O26 and exposure to a farm or nursery. This restricted time period of 5 years was chosen for regression analysis because, firstly, testing for non-O157 serogroups was less widespread in the years immediately after the rollout of PCR testing in 2013. Secondly, the introduction of O26 PCR testing at GBRU improved the surveillance of STEC O26 from 2021 onwards by allowing more timely detection of these cases [18, 21]. Using more recent data for the regression analysis was intended to mitigate the influence of these changes in surveillance on the observed associations. To describe the association between serogroup O26 and severe illness, a case–case study design was used, comparing the risk of severe health outcomes between cases with O26, O157, and all other cases. To describe the association between serogroup O26 and farm or nursery exposure, a retrospective cohort study design was used to compare the risk of exposure to these settings amongst cases with O26, O157, or all other cases. Adjusted risk ratios were calculated using a multivariable binomial regression model for each outcome where possible. A Poisson regression model with robust standard errors was used when the binomial model failed to converge. Models were adjusted for age, sex, and region *a priori.* Age was selected due to the higher risk of attending a farm or nursery, having O26, and having HUS, amongst young children. Region and sex also affect the risk of STEC infection, complications, and exposures [12]. Data analysis was conducted using R version 2022.07.2.

## Results

### Overview of STEC cases and public health incidents, and serogroup O26

**Figure 1.**
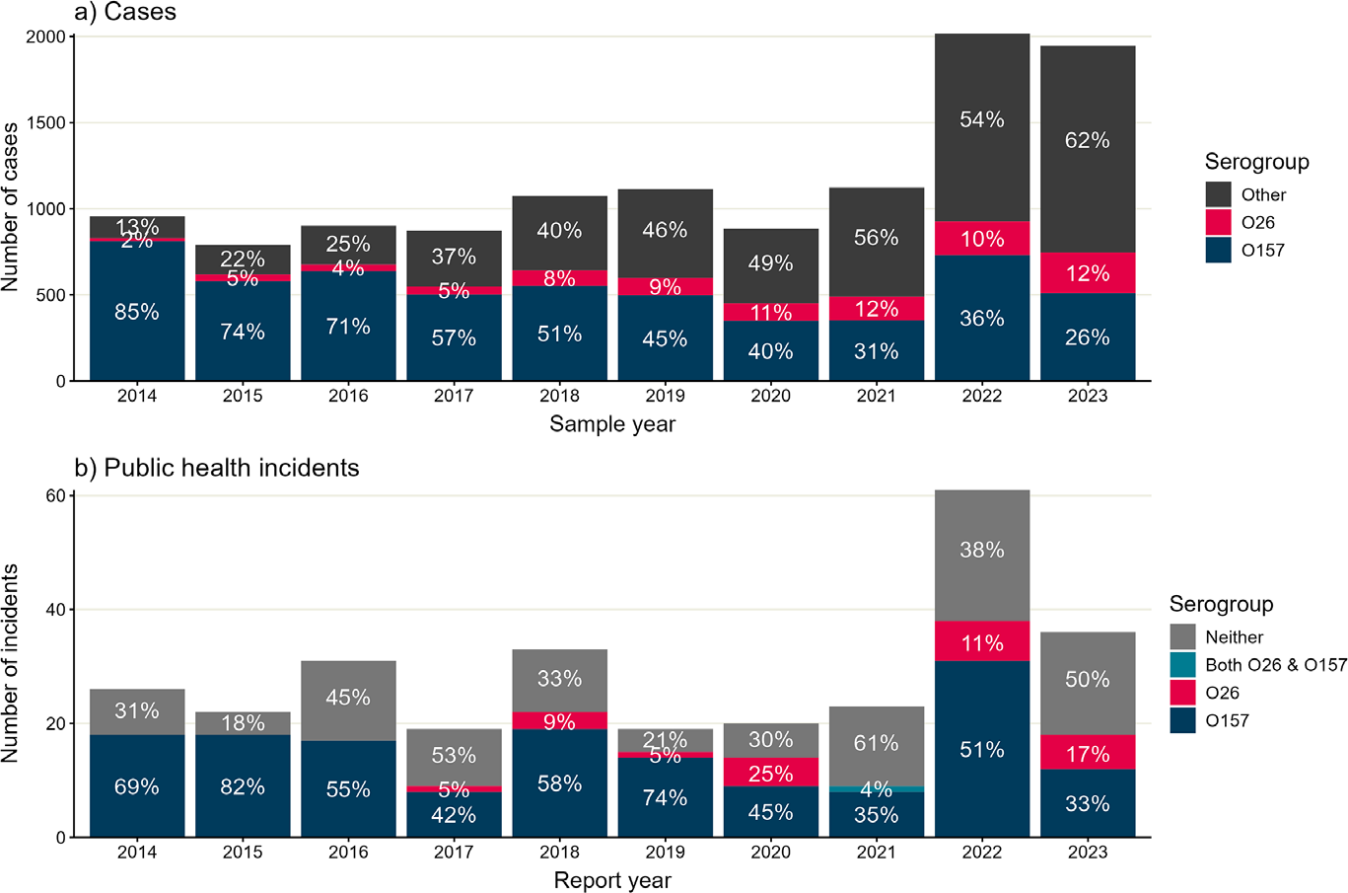
Number of STEC cases and STEC public health incidents by serogroup, England, 2014–2023. (a) Total number of STEC cases by sample year. Only cases with sample date available are included (11,679/12,231). The number of cases of each serogroup are shown in different colours for O157 (navy), O26 (pink), and all other cases (grey). The percentage of cases of each serogroup, each year, are shown with white text labels. (b) Total number of STEC public health incidents by year of report. Showing all 290 incidents with report year available. The number of incidents which mention each serogroup in the descriptive notes are shown in different colours for O157 (navy), O26 (pink), both O26 and O157 (teal, *i.e.*, a mixed serogroup incident involving some cases with O26 and some with O157), or no mention of O26 or O157 (grey). Note that reporting of STEC cases and public health incidents in 2020 and 2021 was affected by the COVID-19 pandemic.

From 1 January 2014 to 31 December 2023, there were 12,231 cases of STEC, including O157 and non-O157, reported to UKHSA overall, increasing from 956 (2014) to 1946 (2023) (Figure 1a). The number of STEC reports remained stable from 2014 to 2021, with a higher number of cases reported in 2022 and 2023.

The most common serogroup reported across the entire period was O157 (47%, 5772/12,231), followed by O26 (9%, 1051/12,231) (Table 1). The number of STEC O157 notifications each year peaked in 2014 and 2022 (which could be associated with large STEC O157 outbreaks), was lowest during the COVID-19 pandemic years of 2020 and 2021, and otherwise remained fairly consistent. The number of non-O157 STEC notifications rose dramatically throughout the decade. The proportion of notifications made up by STEC O157 dropped from 85% (811/956) in 2014 to 26% (511/1946) in 2023, whilst the proportion of notifications with O26 increased sixfold from 2% (19/956) in 2014 to 12% (234/1946) in 2023.

**Table 1.** Characteristics of STEC cases by serogroup, England, 2014–2023

Characteristic	O157	O26	Other
Number of cases	5,772	1,051	5,408
Sex			
Female	3,204 (56%)	598 (57%)	3,096 (57%)
Male	2,566 (44%)	453 (43%)	2,288 (42%)
Unknown	2 (<0.1%)	0 (0%)	24 (0.4%)
Age group			
0–4 years	1,074 (19%)	390 (37%)	579 (11%)
20–29 years	972 (17%)	133 (13%)	775 (14%)
30–39 years	593 (10%)	99 (9.4%)	816 (15%)
10–19 years	719 (12%)	106 (10%)	494 (9.1%)
50–59 years	535 (9.3%)	66 (6.3%)	597 (11%)
40–49 years	456 (7.9%)	47 (4.5%)	603 (11%)
60–69 years	404 (7.0%)	49 (4.7%)	502 (9.3%)
70–79 years	333 (5.8%)	59 (5.6%)	459 (8.5%)
5–9 years	512 (8.9%)	75 (7.1%)	246 (4.5%)
> = 80 years	170 (2.9%)	27 (2.6%)	337 (6.2%)
Unknown	4 (<0.1%)	0 (0%)	0 (0%)
stx type			
stx2 only	3,485 (60%)	318 (30%)	1,516 (28%)
stx1 and stx2	2,246 (39%)	234 (22%)	1,690 (31%)
stx1 only	41 (0.7%)	499 (47%)	2,202 (41%)
Questionnaire completed	5,572 (97%)	834 (79%)	2037 (38%)
No questionnaire	200 (3%)	217 (21%)	3,371 (62%)
Environmental exposures			
Attended farm	868 (16%)	145 (17%)	219 (11%)
Lived in or accessed private farm	378 (6.8%)	50 (6.0%)	94 (4.6%)
Attended nursery	623 (11%)	170 (20%)	156 (7.7%)
Clinical outcomes			
Bloody diarrhoea	3,300 (59%)	378 (45%)	785 (39%)
Admitted to hospital	1758 (32%)	250 (30%)	471 (23%)
Haemolytic uraemic syndrome	145 (2.6%)	73 (8.8%)	73 (3.6%)

Stratified by serogroup (O157, O26, or other), cases were more often female in each serogroup (56–57%), but the age distribution varied, with a greater proportion of O26 cases aged 0–4 years (37%) than O157 (19%) or other cases (11%) (Table 1) (Supplementary Table S1). Bloody diarrhoea was reported for 59% (3,300/5572) of O157 cases, 45% (378/834) of O26 cases, and 39% (785/2037) of other cases, where a questionnaire was available. Hospital admission was highest for O157 (32%, 1758/5,572), followed by O26 (30%, 259/834) and other (23%, 471/2037). HUS was most common for O26 (9%, 73/834), followed by other serogroups (4%, 73/2037) and O157 (3%, 145/5,572). The overall age distribution of STEC cases did not change over time (Supplementary Figure S1).

**Figure 2.**
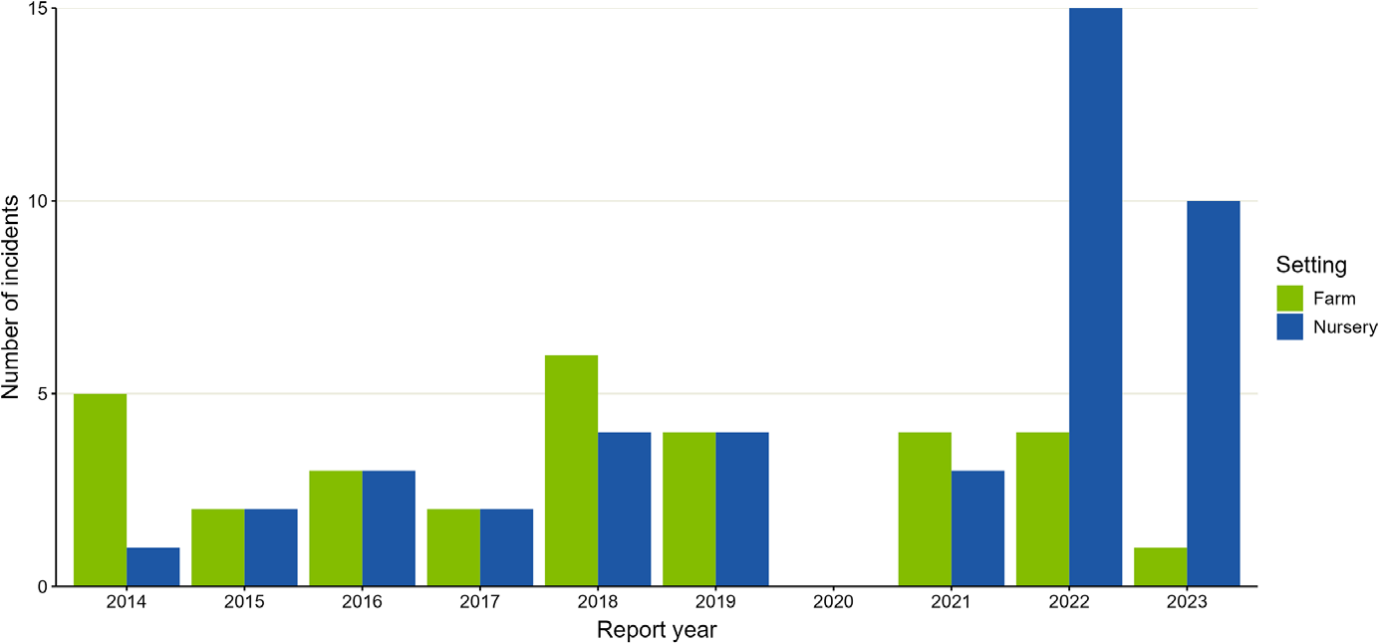
Number of STEC public health incidents based at a farm or a nursery, England, 2014–2023. Total number of STEC public health incidents by year of report into HPZone, which had a farm setting or nursery setting (68/290). The number of incidents with a farm setting is shown in green, and the number of incidents with a nursery setting is shown in blue. Note that reporting of STEC public health incidents in 2020 and 2021 was affected by the COVID-19 pandemic.

From 2014 to 2023, there were 290 public health incidents of STEC reported to UKHSA or its predecessor, Public Health England (PHE), increasing from 26 (2014) to 36 (2023) (Figure 1b) (Supplementary Table S2). Overall, 8% (23/290) of public health incidents referred to STEC O26, increasing from 5% (1/19) (2017) to 17% (6/36) (2023), with no public health incident mentioning STEC O26 on HPZone before 2017 (Figure 2). There were 154 (53%) public health incidents which referred to O157, decreasing from 18 (69%) in 2014, to 12 (33%) in 2023.

### Association between health outcomes and STEC O26

Between 2019 and 2023, cases with STEC O26 had a significantly higher risk of HUS compared to cases with STEC O157 (adjusted risk ratio (aRR) 3.13 (95%CI: 2.18–4.51)) (Table 2). This was also observed for all other non-O26, non-O157 cases compared to STEC O157 (aRR 1.88 (95%CI: 1.25–2.80)). We did not observe a significantly higher risk of bloody diarrhoea or hospital admission in STEC O26 or in other STEC cases compared to STEC O157.

**Table 2.** Risk of health outcomes from STEC O26, compared to STEC O157 (reference group) and to all other cases, England, 2019–2023

	Symptom	%	cRR	P value	aRR*	P value
	Bloody diarrhoea					
	n/N					
O26	286/623	46%	0.80 (0.72–0.88)	< 0.001	0.91 (0.83–0.99)	0.025
Other	534/1,132	47%	0.81 (0.75–0.88)	< 0.001	0.81 (0.75–0.87)	< 0.001
O157	1,365/2,297	59%	Reference group		Reference group	

	Hospital admission					
	n/N	%	cRR	P value	aRR*	P value
O26	201/623	32%	1.02 (0.90–1.16)	0.738	1.14 (1.00–1.30)	0.045
Other	319/1,132	28%	0.89 (0.80–1.00)	0.0449	0.94 (0.84–1.05)	0.304
O157	725/2,297	32%	Reference group		Reference group	

	HUS					
	n/N	%	cRR	P value	aRR*	P value
O26	62/623	10.0%	4.16 (2.92–5.93)	< 0.001	3.13 (2.18–4.51)	< 0.001
Other	40/1,132	3.5%	1.48 (0.98–2.20)	0.0572	1.88 (1.25–2.80)	0.002
O157	55/2,297	2.4%	Reference group		Reference group	

*Note:* Table showing crude risk ratios (cRR) and adjusted risk ratios (aRR) for health outcomes (bloody diarrhoea, hospital admission, and haemolytic uraemic syndrome (HUS)), for STEC O26 compared to STEC O157 (reference group) and to all other STEC cases. *Adjusted for age, sex, and region of the case.

### STEC cases and public health incidents associated with farms and nurseries

From 2014 to 2023, out of 8,443 (69%) STEC cases of all ages and serogroups with a questionnaire available, 1,232 (15%) reported attendance at a farm (either visiting a farm or living at a farm), and 949 (11%) reported attendance at a nursery (either children being cared for at nursery or nursery staff) (Table 1). Overall, 522 (6%) cases reported living in or accessing a private farm. Attendance at farms and nurseries did not change significantly over the decade (Supplementary Figure S2). Stratified by age, 55% (901/1635) of cases aged 0–4 years attended a nursery and 24% (396/1635) attended a farm, compared to 0.7% (48/6804, nurseries) and 12% (835/6804, farms) of cases aged over 4 years. Stratified by serogroup, farm attendance was most commonly reported by O26 cases (17%), followed by O157 (16%), then other cases (11%). Nursery attendance was most common in O26 cases (20%), followed by O157 (11%), then other cases (8%).

Overall, 11% (31/290) of STEC public health incidents had a farm setting, and 15% (44/290) had a nursery setting (Supplementary Table S2). The number of incidents associated with a farm decreased from 5/21 (24%) in 2014 to 1/35 (3%) in 2023; the number of incidents associated with a nursery increased over time from 1/25 (4%) in 2014 to 10/26 (38%) in 2023 (Figure 2). Whilst no farm incidents mentioned O26, 23% (10/44) of nursery incidents referred to O26 (Supplementary Table S2).

### Association between farm and nursery settings and STEC O26

From 2019 to 2023, the adjusted risk of attendance at a farm (aRR 1.14 (0.93–1.38)) was comparable in STEC O26 and STEC O157, and the adjusted risk of nursery attendance (aRR 0.93 (0.90–0.96)) was marginally lower for STEC O26 (Table 3a). There was a slightly lower adjusted risk of farm or nursery attendance amongst cases with non-O26, non-O157 serogroups compared to O157. The crude risk of farm or nursery attendance was higher for STEC O26 compared to STEC O157 and other serogroups. However, when adjusted for demographic variables, including age, an association between STEC O26 and these settings was no longer observed, which may be because a greater proportion of STEC O26 cases are children, and children are more likely to attend farms or nurseries (Table 1, Supplementary Figure S2). To explore the impact of age, crude risk ratios were produced comparing the risk of nursery attendance in different serogroups, stratified into those aged 0–4 years or aged over 5 years. A significant association between STEC O26 and exposure to a farm or nursery, compared to STEC O157, was no longer observed (Table 3b).

**Table 3.** Risk of attendance at a farm or nursery in STEC O26, compared to STEC O157 (reference group) and to all other cases, shown for all cases (a) and stratified by age group (b), England, 2019–2023

(a)						
	Exposure setting	%	cRR	P value	aRR*	P value
	Farm attendance					
	n/N					
O26	112/623	18%	1.29 (1.06–1.56)	0.0108	1.14 (0.93–1.38)	0.199
Other	116/1,132	10%	0.74 (0.60–0.90)	0.00263	0.80 (0.65–0.98)	0.033
O157	320/2,297	14%	Reference group		Reference group	

	Nursery attendance					
	n/N	%	cRR	P value	aRR**	P value
O26	133/623	21%	2.05 (1.69–2.48)	< 0.001	0.93 (0.90–0.96)	0.512
Other	92/1,132	8.1%	0.78 (0.62–0.98)	0.0350	0.93 (0.91–0.95)	0.569
O157	239/2,297	10.4%	Reference group		Reference group	

(b)				
	Exposure setting	%	cRR	P value
	Farm attendance			
	n/N			
Age 0–4 years				
O26	67/244	27%	1.26 (0.95–1.66)	0.104
Other	33/175	19%	0.87 (0.59–1.22)	0.425
O157	86/394	22%	Reference group	
Age 5 or more years				
O26	45/379	12%	0.97 (0.71–1.29)	0.830
Other	83/957	9%	0.71 (0.55–0.89)	0.00431
O157	233/1899	12%	Reference group	

	Nursery attendance			
	n/N	%	cRR	P value
Age 0–4 years				
O26	132/244	54%	0.93 (0.80–1.07)	0.295
Other	86/175	49%	0.84 (0.70–0.99)	0.0501
O157	230/394	58%	Reference group	
Age 5 or more years				
O26	1/379	0.3%	0.56 (0.03–2.95)	0.578
Other	6/957	0.6%	1.32 (0.45–3.66)	0.594
O157	9/1899	0.5%	Reference group	

(a) Table showing crude risk ratios (cRR) and adjusted risk ratios (aRR) for attendance at a farm or at a nursery, for STEC O26 compared to STEC O157 (reference group) and to all other STEC cases. *Binomial regression model adjusted for age, sex, and region. **Poisson regression model with robust standard errors adjusted for age, sex, and region. Poisson regression model used due to non-convergence of the binomial model. (b) Table showing crude risk ratios (cRR) for attendance at a farm or nursery, for STEC O26 compared to STEC O157 (reference group) and to all other STEC cases, stratified by age (aged 0–4 years or aged 5 or more years).

## Discussion

We observed a rise in the overall number of STEC cases and public health incidents in recent years, notably in 2022 and 2023. The consistent number of STEC cases reported from 2014 to 2021 masked an underlying shift in serogroup detection in England, with a decreasing proportion of STEC O157 and increasing STEC non-O157 [31]. Consistent with previous studies using UKHSA surveillance data, we observed a rise in the number of cases and public health incidents associated with STEC O26, as the second most common serogroup after O157 [16, 21].

The increased detection of non-O157 STEC has likely been driven by the implementation of PCR assays for GI infections in local diagnostic laboratories; these assays target the Shiga toxin gene (*stx*) and can detect a wide range of STEC serogroups [16, 31]. An estimated 40% of laboratories in England used PCR diagnostics for STEC in 2023, an increase from three laboratories in 2014 and 20% of laboratories in 2018 [16, 18, 22]. However, although implementation of PCR testing should also improve detection of STEC O157, we did not observe an increase in detections of STEC O157. It is possible that changes in commonly circulating serogroups in the animal reservoir of STEC could contribute to the increasing role of non-O157 in STEC cases [32]. There is evidence of STEC strain replacement events taking place in the UK domestic cattle reservoir over the last 40 years, although these events were characterized by replacement of one lineage of STEC O157 (lineage I/II) with a different STEC O157 lineage (lineage 1c) [33, 34]. To test whether there has been a strain replacement event with non-O157 STEC serogroups, further studies of the UK cattle reservoir would be required. Importantly, decreases in both O157 and non-O157 in 2020 and 2021 are likely to be associated with effects of the COVID-19 pandemic, such as changes in reporting, healthcare-seeking, and exposures. Large outbreaks may also influence the number of STEC cases of each serogroup observed each year; for example, high numbers of STEC O157 reported in 2014 and 2022 are likely attributable to large, food-borne outbreaks in the UK [35, 36].

Despite improvements to STEC diagnostic algorithms in England, it remains difficult to determine the true incidence of non-O157 serogroups [2, 31]. Less than half of diagnostic laboratories conduct *stx*-PCR testing and instead rely on referral of specimens to the reference laboratory for detection of non-O157 STEC. Not all specimens will be referred, particularly for milder cases whose symptoms may have resolved, and any delays in referral and processing reduce the quality of the sample and the chance of detecting STEC. It is therefore likely that the contribution of STEC O26 and other non-O157 STEC to the burden of disease is underestimated.

We found that infection with non-O157 STEC, and particularly STEC O26, was associated with a higher risk of HUS in both children and adults when compared to STEC O157. The relationship between STEC O26 and severe disease outcomes has been described previously and has informed public health guidance in England, prioritizing public health interventions, such as exclusion from nursery school and contact tracing, for cases of O157 or O26 [13, 20, 21, 31]. Our findings add to the body of evidence highlighting STEC O26 as a significant threat to public health for all ages. It is worth noting that these results could be affected by reporting bias. Specimens which test negative for O157 from cases with severe disease are more likely to be referred to the reference laboratory, resulting in limited detection of milder cases of non-O157 STEC, and an inflated association between HUS and non-O157. Widespread testing for non-O157 STEC at local diagnostic laboratories could reduce this bias and provide a greater understanding of the pathogenicity of STEC O26.

We did not observe a change in the patterns of farm or nursery attendance reported by cases of STEC, suggesting that increased exposure to these settings is not responsible for rising STEC incidence. Indeed, the percentage of STEC cases aged 0–4 years that we estimated attended a nursery (55%) is similar to the national estimate of 0–4 -year olds using formal childcare in 2023 (63%) [37]. Regarding STEC public health incidents, a reduction in both farm and nursery-based incidents was observed during the COVID-19 pandemic, likely associated with the closure of nurseries and visitor attractions. After venues had re-opened, we saw an increase in nursery-based STEC incidents, including STEC O26, which would align more closely with the pre-pandemic trend of increasing numbers of nursery- and farm-based STEC outbreaks [33]. However, for sporadic cases, we cannot determine whether attendance at a setting was the likely source of infection, as cases often have multiple exposures, and it is not possible to determine which, if any, was associated with infection. We also cannot rule out unreported changes in exposure settings amongst cases missing questionnaire data [27].

We found no difference in farm or nursery attendance between STEC O26 and STEC O157 after adjusting for risk factors. Whilst the crude risk of farm or nursery attendance was higher in STEC O26 than STEC O157, this was no longer observed after adjusting for or stratifying by age, suggesting an association between STEC O26 and farms or nurseries may be explained by a higher risk of STEC O26 in children. This could be due to increased likelihood of testing for non-O157 in children, who are at higher risk of severe complications, or a genuinely higher incidence of STEC O26 in this age group. Accounting for age differences, our findings provide good evidence that STEC O26 most likely has the same ecological niche and transmission pathways as STEC O157. This is important because we have extensive knowledge of STEC O157, and applying that knowledge to STEC O26 public health incidents will be valuable for outbreak investigations and the management of cases linked to farms and nurseries. Recent STEC O26 outbreaks at farms or nurseries may reflect increased detection of O26 in general and the inherently high-risk nature of these settings for STEC transmission, without an additional risk associated with this serogroup [14, 38]. STEC O26 has frequently been detected in farm animals and their faeces, and attending petting farms or handling farm animals has been shown to increase the risk of infection with O26 and other non-O157 STEC [14, 19, 38–40]. Large outbreaks of STEC O26 associated with nurseries have been reported, and working in childcare has been identified as a risk factor for STEC O26 infection in particular [25, 26, 38]. Our findings suggest STEC O26 transmission at farms and nurseries is as likely as other STEC serogroups, reinforcing the importance of testing for non-O157 STEC amongst exposed cases.

## Conclusions and recommendations

Rising STEC incidence over the last decade has been driven by increasing detection of non-O157 serogroups, and STEC O26 has emerged as the second most common serogroup after O157. STEC O26 was associated with a similar risk of exposure to farm or nursery settings as STEC O157, after adjusting for age, but carried a significantly higher risk of severe morbidity. Understanding the epidemiology of STEC O26 is restricted by limited uptake of non-O157 testing. We recommend the universal implementation of testing for non-O157 STEC at diagnostic laboratories and referral of all positive samples to GBRU. This would enable a more accurate assessment of the impact of O26 and other non-O157 STEC and allow more timely case management prior to the development of severe complications.

## Supporting information

10.1017/S0950268825100654.sm001 Findlater et al. supplementary material Findlater et al. supplementary material

## Data Availability

The data that support this study were collected as part of a public health response and are considered sensitive and not made publicly available. Reasonable requests for access to anonymized data and data dictionary will be considered by the authors on request.

## Supplementary material

The supplementary material for this article can be found at http://doi.org/10.1017/S0950268825100654.
