# Supplementary material for: Increased detection of Shiga toxin-producing Escherichia coli (STEC) O26: Environmental exposures and clinical outcomes, England, 2014–2023
Source: Epidemiol Infect. 2025 Oct 7;153:e123. doi: 10.1017/S0950268825100654 (PMC12529424; doi:10.1017/S0950268825100654)
Supplement: Findlater et al. supplementary material [file S0950268825100654sup001.docx]

***Epidemiology and Infection***

**Rising Shiga toxin-producing Escherichia coli (STEC) O26: environmental exposures and clinical outcomes, England, 2014-2023**

Lucy Findlater, Orlagh Quinn, Amy Douglas, Clare Sawyer, Victoria Hall, Claire Jenkins, Sooria Balasegaram

**Supplementary Material**

**Supplementary Table S1: Characteristics of STEC cases, England, 2014-2023**

| **Characteristic** | **O157** | **O26** | **Other** |
| --- | --- | --- | --- |
| Number of cases | 5772 | 1051 | 5408 |
| Ethnicity |  |  |  |
| White | 3783 (66%) | 563 (54%) | 1125 (21%) |
| Asian or Asian British | 214 (3.7%) | 27 (2.6%) | 120 (2.2%) |
| Other ethnic group | 88 (1.5%) | 22 (2.1%) | 98 (1.8%) |
| Black, Black British, Caribbean or African | 79 (1.2%) | 6 (0.6%) | 39 (0.7%) |
| Mixed or multiple ethnic groups | 54 (0.9%) | 12 (1.1%) | 31 (0.6%) |
| Unknown | 1554 (27%) | 421 (40%) | 3995 (74%) |
| Region |  |  |  |
| South East | 24 (21%) | 36 (23%) | 4 (17%) |
| London | 20 (18%) | 24 (16%) | 4 (17%) |
| South West | 20 (18%) | 15 (9.7%) | 3 (13%) |
| North West | 15 (13%) | 18 (12%) | 4 (17%) |
| West Midlands | 18 (16%) | 12 (7.8%) | 0 (0%) |
| Yorkshire and the Humber | 3 (2.7%) | 14 (9.1%) | 5 (22%) |
| East Midlands | 4 (3.6%) | 15 (9.7%) | 1 (4.3%) |
| North East | 5 (4.5%) | 7 (4.5%) | 2 (8.7%) |
| East of England | 2 (1.8%) | 10 (6.5%) | 0 (0%) |
| National incident | 1 (0.9%) | 3 (1.9%) | 0 (0%) |

Supplementary Table S1: Table displaying characteristics of STEC cases in addition to those described in the manuscript.

**Supplementary Figure S1: Age distribution of cases of STEC, by year of sample, England, 2014-2023**


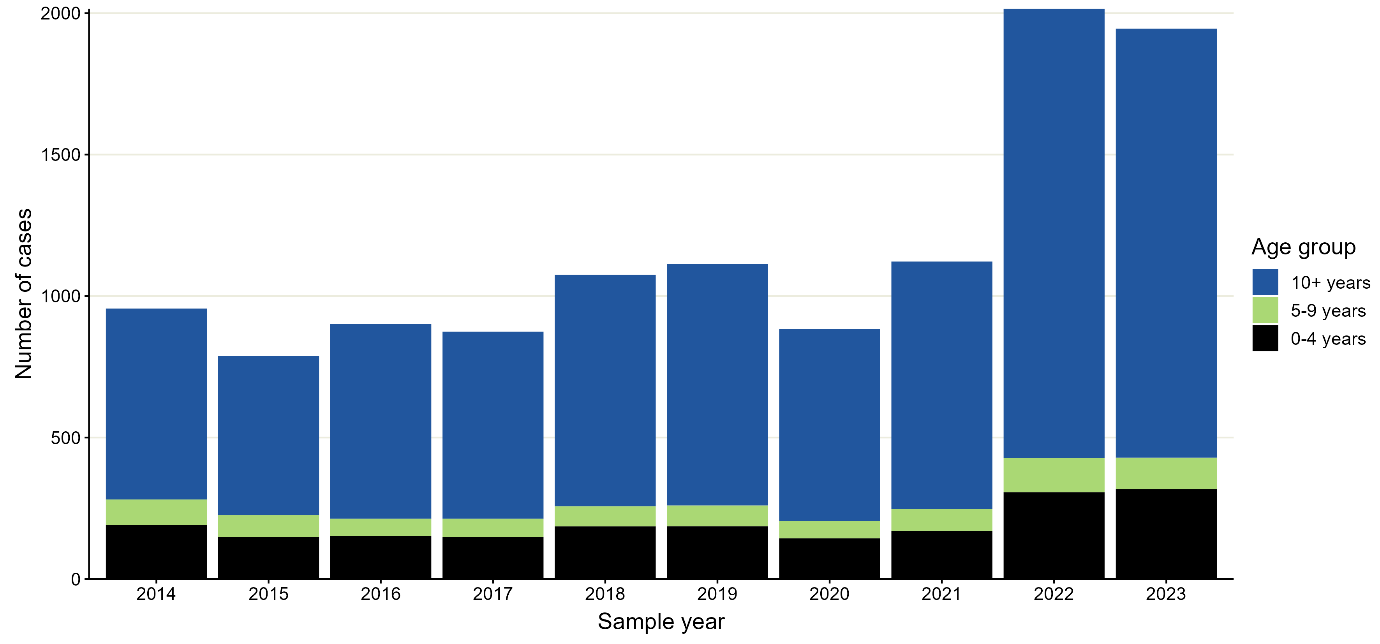


Supplementary Figure S1: Percentage of STEC cases that belong to each age group are shown over time. Cases aged 0-4 years are shown with the black bars, cases aged 5-9 years with the green bars, and cases aged 10 or more years are presented with the blue bars. Showing cases with sample year and age group available (11,675/ 12,231).

**Supplementary Table S2: Characteristics of regional STEC incidents, stratified by serogroup mentioned, England, 2014-2023**

| **Characteristic** | **O157** | **O26** | **Neither O157 nor O26** |
| --- | --- | --- | --- |
| Number of incidents | 154 | 23 | 112 |
| Farm attendance reported | 18 (12%) | 0 (0%) | 12 (11%) |
| Nursery attendance reported | 19 (12%) | 10 (43%) | 15 (13%) |
| Principal context |  |  |  |
| Unknown | 58 (38%) | 3 (13%) | 50 (45%) |
| Household | 27 (18%) | 6 (26%) | 15 (13%) |
| Community | 18 (12%) | 4 (17%) | 13 (12%) |
| Food outlet/restaurant | 12 (7.8%) | 1 (4.3%) | 8 (7.1%) |
| Nursery | 9 (5.8%) | 5 (22%) | 6 (5.4%) |
| Visitor attraction | 13 (8.4%) | 0 (0%) | 5 (4.5%) |
| Other | 5 (3.2%) | 1 (4.3%) | 5 (4.5%) |
| Congregation | 5 (3.2%) | 0 (0%) | 4 (3.6%) |
| School | 2 (1.3%) | 2 (8.7%) | 5 (4.5%) |
| Environmental exposure | 4 (2.6%) | 0 (0%) | 1 (0.9%) |
| Childminder/childcare provision | 1 (0.6%) | 1 (4.3%) | 0 (0%) |
| Region |  |  |  |
| South East | 36 (23%) | 4 (17%) | 24 (21%) |
| London | 24 (16%) | 4 (17%) | 20 (18%) |
| South West | 15 (9.7%) | 3 (13%) | 20 (18%) |
| North West | 18 (12%) | 4 (17%) | 15 (13%) |
| West Midlands | 12 (7.8%) | 0 (0%) | 18 (16%) |
| Yorkshire and the Humber | 14 (9.1%) | 5 (22%) | 3 (2.7%) |
| East Midlands | 15 (9.7%) | 1 (4.3%) | 4 (3.6%) |
| North East | 7 (4.5%) | 2 (8.7%) | 5 (4.5%) |
| East of England | 10 (6.5%) | 0 (0%) | 2 (1.8%) |
| National incident | 3 (1.9%) | 0 (0%) | 1 (0.9%) |

Supplementary Table S1: Showing 289/290 incidents of STEC reported from 2014-2023 by serogroup mentioned. One incident which reported both O157 and O26 cases has not been included in this table. Note that whilst the principal context of an incident may not be categorised by health protection teams as a farm or a nursery, farm or nursery attendance may still be reported by cases within the incident.

**Supplementary Figure S2: Percentage of STEC cases with questionnaire available who reported attendance at a farm or a nursery, stratified by age group, England, 2014-2023**

**
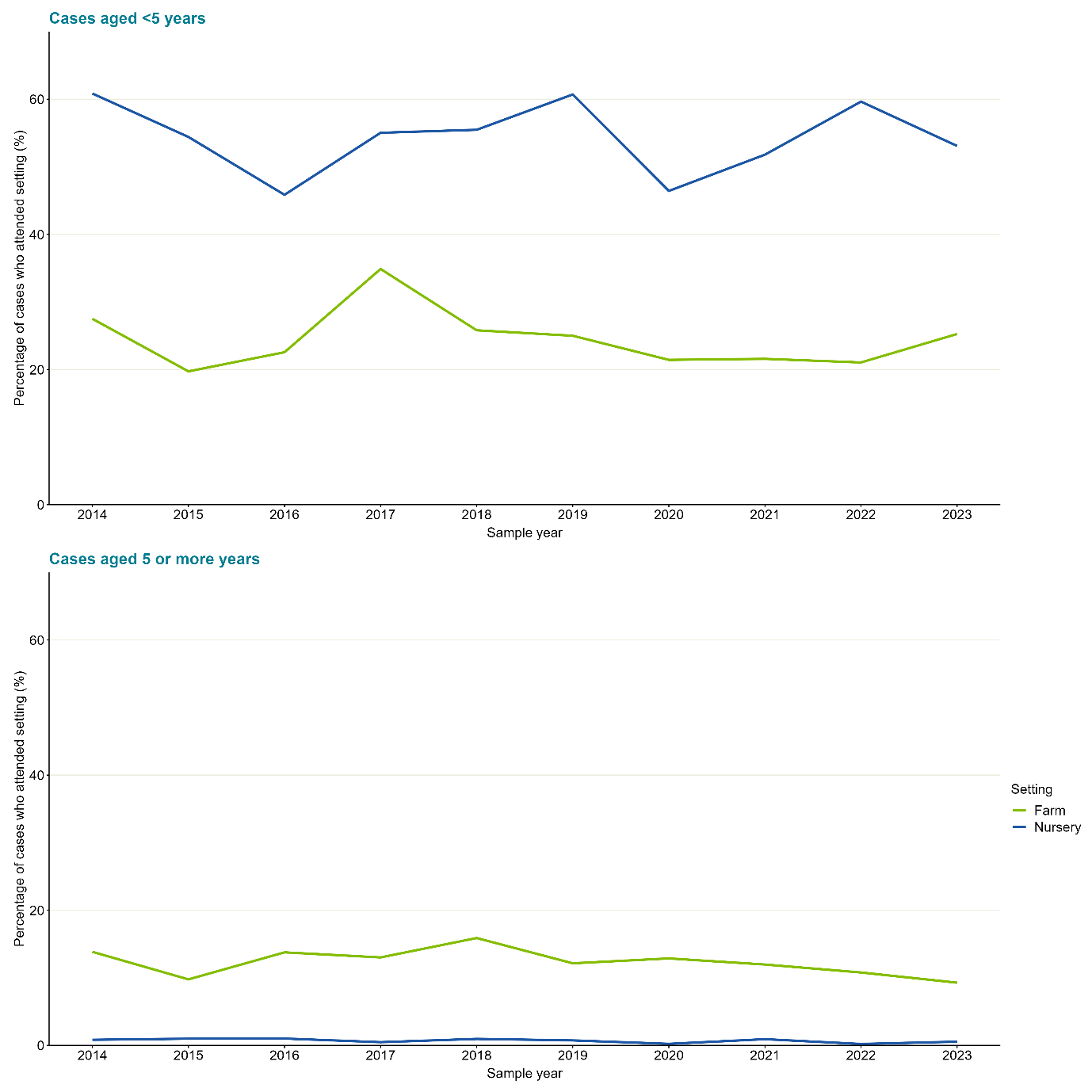
**

Supplementary Figure S3: Showing the percentage of cases with questionnaire available (8443/12,231) who reported attendance at a farm or a nursery in their questionnaire, stratified into age group (either aged fewer than 5 years, or 5 or more years), by sample year. Cases could attend both a farm and a nursery. The percentage of cases who attended a farm is shown with the green line and the percentage of cases who attended a nursery is shown with the blue line.
